# Corn as an Antiperiodic

**Published:** 1858-09

**Authors:** 


CORN AS AN ANTIPERIODIC.—The following is an extract from a letter written from Shanghai to Professor Dunglison, by Dr. D. B. Phillips, of the United States Navy, who is Surgeon, on the steamer "Mississippi," of our squadron at China. Prof. Dunglison has kindly placed it at our disposal:—

"Since writing an article upon the use of the uncooked and fresh meal of Indian-corn as an antiperiodic in intermittent fevers, I have had occasion to use it also in a case of very violent facial neuralgia of a periodic character, which refused to yield either to quinine, arsenic, or other antiperiodics, but rather grew worse. The corn-meal which I used was not fresh and coarsely ground (such as I had found to be best,) nor was it of the yellow kind—which seems to possess the strongest properties—but was finely ground, sifted, white, and evidently rather old. Yet, under all these circumstances, I found it to produce decided benefit, and to stop the paroxysms when everything else failed.

"I think the corn raised in Virginia, North Carolina, Kentucky, and Tennessee, and in similar latitudes, possesses stronger medicinal effects than that found farther north.

"I would be glad if you would give this remedy a trial. My mode of administering it, is to give two large tablespoonsful once every two hours during the day, and it is well to give it in a tumbler of water, stirred up in a gruel or wash. In the hot stages of intermittent fever, it may be given in ice water, and will be found to relieve thirst, and cool the skin sooner and better than any remedy I know."

